# Levels of second hand smoke in pubs and bars by deprivation and food-serving status: a cross-sectional study from North West England

**DOI:** 10.1186/1471-2458-6-42

**Published:** 2006-02-22

**Authors:** Richard Edwards, Christian P Hasselholdt, Kim Hargreaves, Claire Probert, Richard Holford, Judy Hart, Martie Van Tongeren, Adrian FR Watson

**Affiliations:** 1 Department of Public Health, Wellington School of Medicine and Health Sciences, University of Otago, Wellington, New Zealand; 2 Department of Environmental & Geographical Sciences, Manchester Metropolitan University, Oxford Rd, Manchester, UK; 3 East Lancashire Public Health Network, Eagle St, Accrington, UK; 4 North West ASH, Silver St, Bury, UK; 5 Manchester Public Health Development Service, Victoria Mill, Lower VickersStreet, Manchester, UK; 6 Evidence for Population Health, Division of Epidemiology and Health Sciences, University of Manchester, Oxford Rd, Manchester, UK; 7 Centre for Occupational and Environmental Health, Division of Epidemiology and Health Sciences, University of Manchester, Oxford Rd, Manchester, UK

## Abstract

**Background:**

The UK government proposed introducing partial smokefree legislation for England with exemptions for pubs and bars that do not prepare and serve food. We set out to test the hypothesis that pubs from more deprived areas and non food-serving pubs have higher levels of particulate air pollution.

**Methods:**

We conducted a cross sectional study in four mainly urban areas of the North West of England. We recruited a stratified random sample of 64 pubs divided into four groups based on whether their local population was affluent or deprived (using a UK area based deprivation measure), and whether or not they served food. The timing of air quality monitoring stratified to ensure similar distribution of monitoring by day of the week and time of evening between groups. We used a portable air quality monitor to collect fine particle (PM_2.5_) levels over a minimum of 30 minutes in areas where smoking was allowed,, and calculated mean time-time weighted average PM_2.5 _levels.

**Results:**

Mean PM_2.5 _was 285.5 μg/m^3 ^(95% CI 212.7 to 358.3). Mean levels in the four groups were: affluent food-serving pubs (n = 16) 188.1 μg/m^3 ^(95%CI 128.1 to 248.1); affluent non food-serving (n = 16) 186.8 μg/m^3 ^(95%CI 118.9 to 254.3); deprived food-serving (n = 17) 399.4 μg/m^3 ^(95%CI 177.7 to 621.2); and deprived non food-serving (n = 15) 365.7 μg/m^3 ^(195.6 to 535.7). Levels were higher in pubs in deprived communities: mean 383.6 μg/m^3 ^(95% CI 249.2 to 518.0) vs 187.4 μg/m^3 ^(144.8 to 229.9); geometric mean 245.2 μg/m^3 ^vs 151.2 μg/m^3 ^(p = 0.03). There was little difference in particulate levels between food and non food-serving pubs.

**Conclusion:**

This study adds to the evidence that the UK government’s proposals for partial smokefree legislation in England would offer the least protection to the most heavily exposed group - bar workers and customers in non food-serving pubs in deprived areas. The results suggest these proposals would work against the UK government’s stated aim to reduce health inequalities.

## Background

Second hand smoke (SHS) exposure causes of a range of serious adverse health effects in children, adults and pregnant women [1-3]. In the UK it is estimated that domestic exposure to secondhand smoke causes at least 3,600 deaths annually, while workplace exposure accounts for around 700 deaths per year

Internationally, the introduction of legislation to ensure workplaces and other public places are smokefree is a key current public health policy issue. In many countries and states, such as Ireland, New York, New Zealand and Norway, comprehensive smokefree legislation has been introduced. In the UK, Scotland and Northern Ireland propose to do the same. However, the UK government in its public health White Paper *Choosing Health *proposed introducing partial smokefree legislation for England with exemptions including for pubs and bars that do not prepare and serve food, and private members clubs [4].

A number of groups have criticised the logic of the partial exemption of pubs and bars as studies have shown that the highest levels of SHS exposure occur in pubs and bars [5-7]. The policy may also maintain or even increase differentials in ill health due to smoking as there is evidence that pubs and bars in more deprived areas are less likely to serve food and more likely to allow unrestricted smoking [8,9].

We set out to test hypothesis that pubs and bars from more deprived areas and non food-serving pubs have higher levels of particulate air pollution.

## Methods

### Setting and sampling

The study was performed in four wholly or mainly urban Local Authority areas across the north west of England – Burnley, Bury, Blackburn with Darwen, and Manchester. Each includes areas with relatively high levels of deprivation. The sampling frame for the study was the bars and pubs from the four study areas which had previously participated in a survey of North West hospitality premises [8]. We contacted pubs by letter and excluded those that did not wish to take part from the sampling frame. We also excluded town/city centre pubs as the area-based deprivation score is less likely to be representative of the socio-economic status of its customers. Finally, we excluded a number of pubs from the study during the data collection period as these were deemed to be too unsafe by the researchers.

Pubs were allocated an area-based deprivation score (Index of Multiple Deprivation [IMD] 2004 [10]) by mapping their postcode to the Census Super Output Area. Super Output Areas were categorised into deprivation quintiles (IMD 1 – 5) for all Super Output Areas across the North West. We identified four categories of pubs and bars. Firstly, we dichotomised pubs into affluent (IMD 1–3) and deprived (IMD 4 or 5). Secondly, we divided pubs into those which did and did not prepare and serve food based on their response in the earlier survey [8]. We aimed to recruit four pubs from each of the four categories in each study area (64 pubs in total). In order to maximise the contrast between the groups, for each study area and pub category we sampled randomly first from quintile 5 or 1 pubs in the sampling frame, then if necessary from quintile 4 or 2, and finally from quintile 3, stopping once four pubs plus 3–4 reserves were identified.

### Data collection

We aimed to perform air quality measurements in four pubs per evening, one from each category, visited in sequence. To ensure a range of measurements we sampled on four separate days of the week (Tuesday-Friday). Pub occupancy may vary by day of the week and time of night. Therefore, we included a pub from each category on every evening and varied the order of monitoring the pubs so that a pub from each of the four categories was monitored once first, once second, once third and once fourth within each of the four study areas. Occasionally, to minimise transport time, the order of visiting pubs or choice of pubs had to be slightly modified from this plan.

Data collection was carried out using portable air quality monitors according to a protocol modified from one developed for a US study [11]. Pubs were visited unannounced by two or more investigators. The investigators carried a battery operated real time aerosol monitor (*TSI SidePak AM510 Personal Aerosol Monitor *– TSI, Inc., St. Paul, USA) in a small bag. The monitor was zero calibrated each evening. It was fitted with a 2.5 μm impactor to sample and record the average levels of RSP (PM_2.5_) over one minute periods. The air flow rate was 1.7 l/min. A length of Tygon™ tubing was attached to the inlet of the SidePak, with the other end left protruding outside the bag. The recorded measurements were downloaded to a PC for analysis. A calibration factor of 0.32 was applied based on calibration work with a ThermoMIE personalDataRAM model pDR-1200 real-time aerosol monitor (ThermoAndersen, Inc., Smyrna, GA, USA). This type of monitor had been used in a previous similar study in Delaware and has been calibrated against standard pump-and-filter gravimetric methods [12].

At each venue we identified the busiest room of the pub where smoking was allowed, and selected a central area for monitoring, away from any open external doors or windows, kitchen areas, and not in the immediate vicinity (< 1 metre) of anyone smoking (unless this were not possible). Smoke free areas or rooms were avoided. To avoid affecting occupants' behaviour, investigators acted as normal customers and bought a drink before moving to the sampling area.

The bag was carried or placed on a seat or table wherever possible to sample the ambient air. We recorded the place of monitoring within the venue and time of entry. Recording occurred for at least 30 minutes and usually lasted about 30–35 minutes. We recorded whether or not special events likely to affect occupancy, such as quiz nights, were taking place.

### Analysis

We calculated a time-weighted average PM_2.5 _level for each venue, and the mean PM_2.5 _levels by the four pub types and by deprivation and food-serving categories separately. PM_2.5 _levels generally had a highly positively skewed distribution, so significance tests were performed using log transformed data, and geometric means are also reported where appropriate.

### Ethics

Ethical approval was not required for this study as no measurements were made on human subjects.

## Results

Of 402 pubs in the sampling frame, 71% were in deprived areas (IMD4-5). Only nine refused to take part. Monitoring was completed in 64 pubs, 16 in each study area. Equal numbers from each pub category were sampled on each evening except for one night in Burnley when a food-serving pub from a deprived area was substituted for a non food-serving pub.

The mean time of starting monitoring by type of pub was as follows: affluent food-serving 8.58 pm (range 7.15 to 10.50 pm,); affluent non food-serving 9.17 pm (range 7.50 to 10.40 pm); deprived food-serving 8.55 pm (range 7.45 to 10.25 pm); deprived non food-serving 9.08 pm (range 7.36 to 10.20 pm); and all affluent 9.08 pm (range 7.15 to 10.50 pm) and all deprived 9.01 pm (range 7.36 to 10.25 pm).

**Figure 1:**
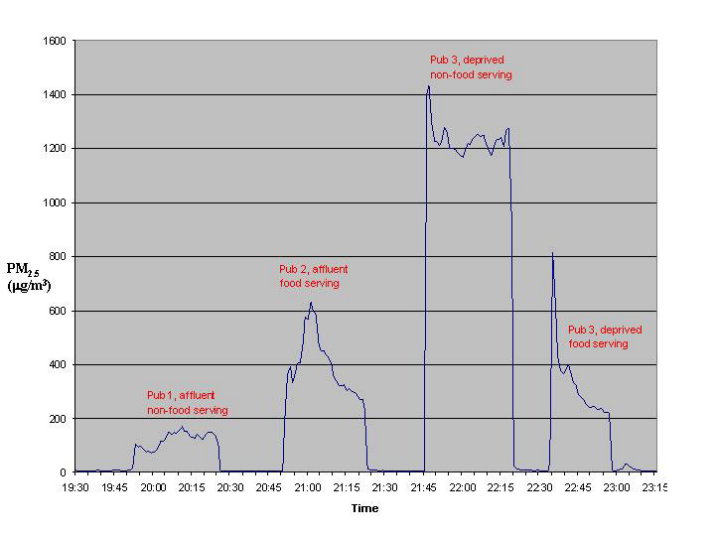
Fine particle levels by pub category on one night's monitoring, September 2005.

Figure 1 shows a typical plot from a single night's monitoring. It shows the dramatic increase in fine particle levels seen as we moved from the external ambient air and entered each pub. It also shows the variability in levels between pubs with the highest levels seen on this evening in the third pub, which was a non food-serving pub from a deprived area.

The mean level of PM_2.5 _across all venues was 285.5 (95% CI 212.7 to 358.3) μg/m^3^. Levels were highest on Thursdays (mean PM_2.5 _= 356.7 μg/m^3^) and lowest on Tuesdays (mean PM_2.5 _= 187.3 μg/m^3^). Mean levels varied by order of visit as follows: 287.3 μg/m^3 ^for the first, 209.5 μg/m^3 ^for the second, 311.6 μg/m^3 ^for the third, and 333.4 μg/m^3 ^for the final visit.

**Figure 2:**
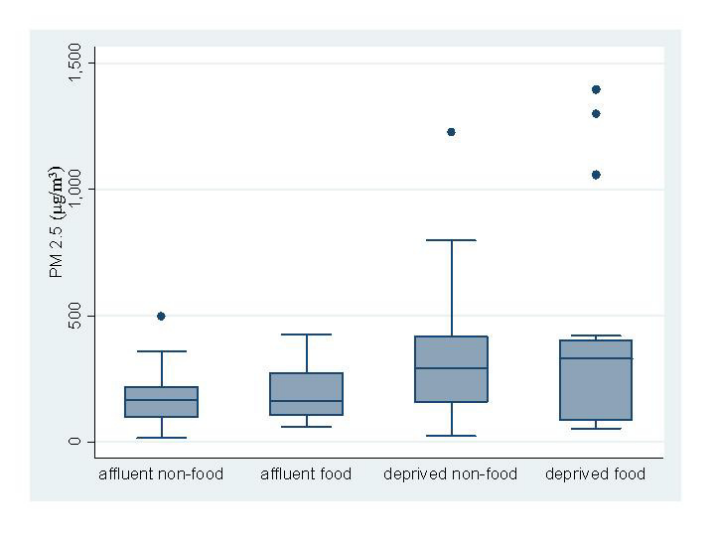
Box plots of fine particle levels by category of pub in μg/m^3^.

Table 1 shows the mean, median and range of PM_2.5 _levels by pub category. Figure 2 shows these data graphically. The geometric mean for time weighted averages of PM_2.5 _were higher in pubs in deprived communities (245.2 μg/m^3^, 95%CI 169.9 to 353.9 μg/m^3^) than in pubs in more affluent areas (151.2 μg/m^3^; 95%CI 116.4 to 196.4 μg/m^3^), p = 0.03. There was little difference in levels between food and non food-serving pubs (difference in geometric means = 2.4 μg/m^3^, p = 0.96).

## Discussion

The English Public Health White Paper proposed to exempt non food-serving pubs and bars from the  smokefree legislation. This study was designed to investigate air quality in pubs in relation to this proposal, and the degree to which air quality varied by deprivation. This is the first such investigation that we are aware of in the UK or elsewhere. We found that levels of fine particles were about twice as high in pubs situated in more deprived areas, but there was little difference between food and non food-serving pubs. Our measurements spanned a range of days and times, and included relatively quiet times early on in midweek evenings. We excluded Saturday nights, when occupancy and hence smoking levels are likely to be at their greatest. Despite this four pubs still had extremely high levels of PM_2.5._

Fine particle levels have been used in previous studies of air quality in the workplace, including in the hospitality trade [11,12]. We found extremely high levels of PM_2.5 _in these hospitality venues, of the same order as those found in similar studies of 20 hospitality venues from New York (mean 324 μg/m^3^), [11] and eight hospitality venues from Delaware (mean 231 μg/m^3^) [12].

Levels of PM_2.5 _observed were much higher than seen in heavily trafficked urban areas. For example, mean ambient PM_2.5 _levels in central London (Marylebone Road) in September 2005 were 23.5 μg/m^3^, 24.6 μg/m^3 ^between 7 and 12 pm. [13] In the UK, there is no air quality standard for PM_2.5, _though one may be introduced soon. In the USA, the Environmental Protection Agency (EPA) uses an air quality standard for ambient air, one component of which is the mean PM_2.5 _level over a 24 hour period [14]. Levels over 65, 150 and 250 μg/m^3 ^are labelled by the EPA as 'unhealthy', 'very unhealthy' and 'hazardous' respectively [15]. Direct comparisons are only a rough guide as the EPA Air Quality Index refers to time weighted averages over a 24 hour period. However, using the EPA categorisation, air quality was unhealthy or worse in 57 out of 64 (89%) of the pubs, and was hazardous in 26 (41%). Of the pubs with hazardous levels (> 250 μg/m^3^), 19 were in deprived areas. All the pubs with very high levels – two 500–999 μg/m^3^and four > 1000 μg/m^3 ^– were in deprived areas.

A limitation of the study was the lack of information on ventilation in the participating pubs. However, the data represent current figures from real world settings and should therefore be representative of air quality experienced by customers and staff across a range of UK pubs. As noted above, the lack of sampling on Saturday nights means that we may have missed periods when air quality is poorest. The very high levels of over 1000 μg/m^3 ^seen in four pubs in our sample show how poor air quality can be in the smokiest venues.

## Conclusion

In conclusion, we found high levels of fine particles in 64 pubs from across the north west of England. Air quality was poorest in pubs situated in more deprived areas. Most (86%) pubs in the sampling frame from the four study areas were in IMD deprivation quintiles 3 to 5, and 71% were in quintiles 4 or 5. The equivalent figures were 82% and 65% respectively in North West pubs from 15 Local Authority areas [8]. This suggests that the higher levels of SHS will be found in most pubs in the North West. Furthermore, the North West survey found that pubs which don't serve food, which will be exempt from the English Public Health White Paper smokefree legislation, will be concentrated in deprived areas [8]. Therefore, this study adds to the evidence that the UK government's proposals for partial smokefree legislation in the UK would offer the least protection to the most heavily exposed group – bar workers and customers in non food-serving pubs in deprived areas. This finding, together with the evidence that smokefree public places and workplaces are effective policies for reducing overall smoking prevalence, [16] suggest that the proposals for partial smokefree legislation are contrary to the UK government's stated aim to reduce health inequalities.

## Competing interests

No specific funding was allocated to this study. Richard Edwards was unpaid chair of NW ASH and Claire Probert paid campaign director of NW ASH at the time of the study. All other authors have no competing interests to declare.

## Authors' contributions

All authors contributed to developing the original idea for the study and the study protocol. Data collection was carried out principally by CPH, KH, CP and RH with additional help from the other authors. The dataset for analysis was prepared by RE, CPH and AFRW. Analysis was carried out mainly by RE with additional suggestions from AFRW and MvT. The paper was drafted by RE. All authors contributed comments on the first and subsequent drafts, and approved the final version for submission. RE is the guarantor of the study.

## Pre-publication history

The pre-publication history for this paper can be accessed here:



## Figures and Tables

**Table 1:** Fine particle (PM_2.5_) levels by pub category in μg/m^3^

	Mean (95% CI)	Median	Range
Affluent food-serving pubs (n = 16)	188.1 (128.1 to 248.1)	160.3	59.3–424.4
Affluent non food pubs (n = 16)	186.6 (118.9 to 254.3)	163.7	14.9–497.4
Deprived food-serving pubs (n = 17)	399.4 (177.7 to 621.2)	329.8	54.1–1395.1
Deprived non food pubs (n = 15)	365.7 (195.6 to 535.7)	290.3	23.5–1226.6
All affluent pubs (n = 32)	187.4 (144.8 to 229.9)	161.3	14.9–497.4
All deprived pubs (n = 32)	383.6 (249.2 to 518.0)	312.4	23.5–1395.1
All food-serving pubs (n = 33)	293.6 (172.1 to 415.2)	166.7	54.1–1395.1
All non food pubs (n = 31)	277.3 (189.8 to 364.8)	216.6	14.9–1226.6
